# Meal patterns associated with energy intake in people with obesity

**DOI:** 10.1017/S0007114521002580

**Published:** 2022-07-28

**Authors:** Cathrine Horn, Johnny Laupsa-Borge, Amanda I. O. Andersen, Laurence Dyer, Ingrid Revheim, Trine Leikanger, Nicole Tandrevold Næsheim, Inghild Storås, Kristine Kjerpeseth Johannessen, Gunnar Mellgren, Jutta Dierkes, Simon N. Dankel

**Affiliations:** 1 Mohn Nutrition Research Laboratory, Centre for Nutrition, Department of Clinical Science, University of Bergen, Bergen, Norway; 2 Mohn Nutrition Research Laboratory, Centre for Nutrition, Department of Clinical Medicine, University of Bergen, Bergen, Norway; 3 Hormone Laboratory, Department of Medical Biochemistry and Pharmacology, Haukeland University Hospital, Bergen, Norway

**Keywords:** Meal patterns, Dietary patterns, Energy intake, Obesity, Eating frequency

## Abstract

It is widely assumed that people with obesity have several common eating patterns, including breakfast skipping, eating during the night and high fast-food consumption. However, differences in individual meal and dietary patterns may be crucial to optimising obesity treatment. Therefore, we investigated the inter-individual variation in eating patterns, hypothesising that individuals with obesity show different dietary and meal patterns, and that these associate with self-reported energy intake (rEI) and/or anthropometric measures. Cross-sectional data from 192 participants (aged 20–55 years) with obesity, including 6 d of weighed food records, were analysed. Meal patterns and dietary patterns were derived using exploratory hierarchical cluster analysis and k-means cluster analysis, respectively. Five clear meal patterns were found based on the time-of-day with the highest mean rEI. The daily rEI was highest among ‘midnight-eaters’ (10 669 (sd 2301) kJ), and significantly (*P* < 0·05) higher than ‘dinner-eaters’ (8619 (sd 2301) kJ), ‘lunch-eaters’ (8703 (sd 2176) kJ) and ‘supper-eaters’ (8786 (sd 1925) kJ), but not ‘regular-eaters’ (9749 (sd 2720) kJ). Despite differences of up to 2050 kJ between meal patterns, there were no significant differences in anthropometric measures or physical activity level (PAL). Four dietary patterns were also found with significant differences in intake of specific food groups, but without significant differences in anthropometry, PAL or rEI. Our data highlight meal timing as a determinant of individual energy intake in people with obesity. The study supports the importance of considering a person’s specific meal pattern, with possible implications for more person-focused guidelines and targeted advice.

Since 1975, the prevalence of obesity has tripled worldwide and today over 650 million people have obesity, accounting for 13 % of the world’s adult population^(4)^. Obesity is a chronic disease and a metabolic risk factor of non-communicable diseases such as CVD, type 2 diabetes, obstructive sleep apnoea and several types of cancer^(5)^. Obesity is also associated with hypertension, elevated cholesterol levels and dyslipidaemia^(6)^. The burden of disease caused by obesity is still rising^(4)^.

Obesity is caused by a chronic imbalance between energy intake and energy expenditure, resulting in excessive fat accumulation^(7)^. This imbalance results from a complex interplay between non-genetic environmental factors (e.g. energy intake and physical activity) and individual genetic predisposition, interacting through epigenetic mechanisms^(8)^. Diet is a modifiable factor, and strategies to avoid excess total energy intake are considered key for preventing and treating obesity^(9)^.

Daily energy intake is determined by food choices and eating behaviour, influenced by a combination of physiological (e.g. appetite and hunger feeling)^(10)^, psychological (e.g. perceived appropriateness or stress)^(11,12)^ and environmental (e.g. portion size, availability, price, or convenience)^(13–15)^ factors. Despite a complex aetiology, previous studies examining obesity and dietary intake have often focused on a single or a few nutrients or foods. The single nutrient approach in nutritional epidemiology may be insufficient in addressing the impact of overall diet on obesity, resulting in inconsistent findings^(16,17)^.

Beyond the traditional single-nutrient approach, the importance of understanding more complex dietary patterns has gained interest in recent years^(18)^. This shift of focus may be seen in connection with a change in the diet-related global disease burden, changing from undernutrition and deficiency of specific nutrients to non-communicable diseases^(5,19)^. Dietary pattern analysis allows for examination of the overall diet, including complex combinations of nutrients and food consumed together, and captures a greater picture of food choices and eating behaviours^(20)^.

However, results from studies of dietary patterns in persons with obesity are inconsistent. When assessing longitudinal changes in anthropometric measures, dietary patterns rich in high-fibre foods and reduced-fat dairy products have been associated with lesser gains in BMI^(21)^, while others conclude that no specific dietary pattern can predict changes in BMI^(22)^.

Recent hypotheses suggest that also meal timing and associated eating behaviour may influence weight regulation, and that timing of food intake is associated with the development of obesity^(23–25)^. Additionally, the distribution of energy intake throughout the day may have effects on metabolic health, such as when meal patterns conflict with internal circadian clocks (chronotype)^(24,26)^.

To our knowledge, few studies report combined data for meal patterns and dietary patterns, as previous studies of meal patterns in people with obesity focus primarily on the association between meal size^(27)^, timing^(28)^ and frequency^(29)^, and less on the intake of nutrients and food groups. The present study, therefore, examined the food intake profiles of a population with obesity, including the timing of food intake. In particular, we conducted a parallel analysis of dietary and meal patterns, hypothesising that people with obesity have distinct dietary and meal patterns and that these differentially associate with energetic intake and/or anthropometric measures. Insight into specific dietary and meal patterns in a population with obesity may contribute to findings of clinical relevance for more targeted personalised intervention strategies.

## Methods

### Participants and study design

Data included in this exploratory analysis were collected before randomisation in a randomised controlled trial investigating the effects of dietary carbohydrates on internal body fat in men and women with obesity (Clinical Trials Identifier NCT03401970). This study was conducted according to the guidelines laid down in the Declaration of Helsinki, and all procedures involving human subjects were approved by the Regional Ethics Committee in Western Norway (Effects of carbohydrate quality and quantity in women and men with obesity, 2017/621/REK West). Written informed consent was obtained from all subjects. Participants were recruited through local newspaper advertisements, radio broadcasts and social media (including advertisement on Facebook), in and surrounding Bergen, Norway, between November 2017 and June 2019.

One hundred and ninety-two male and female subjects with abdominal obesity (BMI ≥ 30 kg/m^2^) and/or waist circumference ≥ 102 cm for males and 88 cm for females were included in the randomised controlled trial, and the baseline data for these are analysed in the present study. Inclusion criteria were age 20–55 years and < 5 % change in body weight within the last 2 months. Exclusion criteria included smoking, known food allergies, alcohol consumption of > 2 alcohol units per day (1 unit being defined as 12 g of alcohol according to the definition of a unit used in the Nordic countries^(30)^), recent surgical or antibiotics treatment during the past 2 months, use of statins and/or diabetes medication, severe diseases, including chronic inflammatory bowel disease, pregnancy, breast-feeding and post-menopause.

### Dietary recordings

Participants conducted 6 d of weighed food records, including four weekdays and two weekend days. Each participant received individually tailored recording days allowing for Fridays in some cases to be considered a weekend day. Therefore, 5/6 or 6/6 reporting days were consecutive days. Participants were asked to record consumption of food and beverages, including all meals and snacks, the weight and amount of all consumed ingredients and products, the time of intake and any additional comments on their food intake. Each participant received a personal user ID in an online dietary recording system (www.diett.no; operated by Dietika AS) to submit daily food consumption. Before data collection, the participants took part in training classes on the use of the dietary recording system and received a digital kitchen scale. The participants were explicitly asked to not change their dietary habits during the period of data collection, and the scientific value of honest and complete records was strongly emphasised.

Following the export of the dietary recording data from www.diett.no, nutritional content was calculated largely based on the latest update of the official Norwegian Food Composition Table^(31)^, or the nutrient declarations provided by the producer/retailer. Values from international databases were used (Danish or US food composition tables, three and eight food items, respectively) when Norwegian data were not available. These comprehensive data were merged into a database using the application FileMaker Pro 18 Advanced (Claris International Inc.) where over 1000 individual products were added. In this database, all recorded consumed ingredients and products were categorised into fifteen food groups and five beverage groups to allow further investigation of the meal and dietary patterns (Table 1). Manual data integrity checks were performed for quality assurance of dietary recordings to help identify and correct possible errors caused by misunderstandings of responders, misinterpretations or keying entry errors. Altogether, the database contained 2210 food and beverage items.

**Table 1.** Recorded food and beverage items categorised into twenty food groups and beverages groups

Food and beverage groups	Food and beverage items
Bread and cereal products	Multi-grain muesli, roasted granola, oatmeal (with fruits and nuts), cereal, cereal-based energy bars, bran flakes, sugary cereals (e.g. Cheerios), crispbread, crispbread (rye), crispbread (oats), crackers, bread, wholemeal bread, rolls, sandwich baguettes, baguettes, bread, rolls, oat bread, buns, rice porridge, oatmeal (prepared), porridge
Rice, pasta	Pasta, rice
Sugary foods	Chocolate cake, cake with cream, brownies, cheesecake, brownie cookies, brownies with chocolate icing, chocolate muffins, apple cake, ice cream (cream-based), ice cream (juice-based), rice pudding, chocolate mousse, chocolate sauce, vanilla sauce, milk chocolate, chocolate bar, licorice, milk chocolate with filling, cookies, biscuits, chocolate cookies, oat biscuits, jaffa cakes, cinnamon rolls, waffles, buns with icing, sweet roll (custard-filled), sweeteners, honey, sugar, brown sugar, nut-based chocolate spreads
Convenience foods	Pizza, ready meals, tacos, crisps, nachos
Cheese	White cheese, cream cheese, brown cheese (brunost)
Dairy products	Light cream, cream, sour cream dressing, creme fraiche, coffee cream, dairy butter, margarine, yogurt, yogurt (flavoured), yogurt (flavoured with toppings)
Meat	Chicken slices, beef mince, bacon pie, meat sausage, chicken fillet with spices, snack ham (diced), liver paste, ground beef patties, chicken breast fillet, chicken thighs, chicken (whole), ham, mutton sausage, liver paste, salami, lasagna, hamburger (home), hamburger (fast food), stew
Seafood	Cod roe, smoked salmon, mackerel in tomato sauce, sardines in tomato sauce, salmon fillet, fish pasta bake, sushi, fish soup, crabsticks, shrimp
Vegetables	Cucumber, red peppers, iceberg lettuce, carrot, red onion, white onion, tomato, broccoli, maize (canned), potato (boiled), potato puree, French fries, Greek salad (feta, olive), mixed bean stew, salad
Fruit	Banana, orange, blueberries, apple, grapes, strawberry, avocado, pear, lemon
Egg	Eggs (boiled), eggs (scrambled), eggs (fried)
Nuts	Walnuts, peanut butter, peanuts, almonds, chia seeds, hazelnuts, nut, and fruit mix
Cooking oils	Olive oil, rapeseed oil, coconut oil
Sauces and dressings	Mayonnaise, light mayonnaise, remoulade, salad dressing, taco seasoning mix, powdered mashed potatoes, bearnaise sauce, gravy (onion), gravy (meat), Mexican-style casserole with rice (powdered), tomato sauce (powder), pesto
Pulses	Sugar peas, chickpeas, baked beans (in tomato sauce), brown beans, green peas, peas, white beans
Alcohol	Red wine, beer, white wine, draft beer, sparkling rosé, liquor, ‘light’ beer
Coffee and tea	Coffee, carbonated water, black tea, fruit tea, caffe latte, green tea
Milk and milk-based drinks	Milk (full-fat), milk (skimmed), chocolate milk
Sweet drinks/juices (energetic)	Coca-Cola, soda, orange juice, apple juice
Sweet drinks/juices (non-energetic)	Coca-Cola zero, soda with artificial sweeteners

### Physical activity level and BMR

Concurrent with the first 3-d weighed food records, participants also recorded all daily life activities and sports for three consecutive 24-h periods to assess their daily level of physical activity. The frequency, duration and intensity for all daily life activities and sports were recorded in the same online system as used for dietary recordings. The recordings were used to estimate a physical activity level (PAL) for each participant based on the sum of estimated energy expenditure for each recorded activity and their associated metabolic equivalent values^(32)^ divided by 24 h. Estimated BMR was calculated with the Mifflin-St Jeor equation validated for individuals with obesity^(33)^.

### Body composition and clinical variables

Height was measured in the upright position with the Frankfort plane horizontal, using a portable stadiometer (Seca 217, Seca GmbH & Co. KG). Body weight was measured with a Class III approved calibrated scale (Seca 877, Seca GmbH & Co. KG) to the nearest 100 g in light clothing without shoes. Waist circumference was measured halfway between the point of the lowest rib and the iliac crest and was repeated three times. The average of the two last measurements was recorded. Body composition was measured by a segmental multifrequency bioelectrical impedance analyser (Seca mBCA 514, Seca GmbH & Co. KG) to assess body fat mass. The measurements were conducted following the device manufacturers’ instructions.

### Statistical analyses

Data are presented as raw unadjusted means and standard deviations unless otherwise stated. Statistical analyses were performed with R, version 4.0.3 (https://www.R-project.org). All inferential tests were two-tailed with a nominal alpha level of 0·05.

To examine meal patterns, we used Ward’s hierarchical cluster method^(34)^ to analyse participants based on total energy intake during different time intervals. We chose hierarchical clustering due to its flexibility when analysing time-dependent data. We categorised and named all eating occasions into six 4-h periods based on the registered time of intake: 04.00 ± 2 h (early morning meal), 08.00 ± 2 h (breakfast), 12.00 ± 2 h (lunch), 16.00 ± 2 h (dinner), 20.00 ± 2 h (supper) and 00.00 ± 2 h (midnight meal). For each participant, we calculated the mean daily energy percentage intake in each time interval across all six recording days and submitted these values to clustering.

When individual time measurements were missing, imputation was performed in R to assign missing time values to items that were eaten within the same meal period. For example, if a participant had recorded four entries for lunch on a specific day but had only recorded the time measurement for a single one of these items, the remaining three items were assigned to the same 4-h time period. For each participant-day combination, imputation was performed, and where imputation could not be performed to generate a complete time-record spanning 24 h, the day was excluded from the analysis. Participants who had no days with a full and valid time record were fully excluded from the hierarchical cluster analysis.

We generated hierarchical cluster solutions for both male and female participants separately, but similar results lead us to retain a cluster solution that included both male and female participants, allowing maximal statistical power.

Using k-means cluster analysis^(35)^, we examined dietary patterns within the data based on the variation of the mean daily intake in g of the food and beverage groups. We chose k-means clustering using daily intake in g rather than energy intake from the specific food and beverage groups due to the widespread use of low-energy products in the dietary recordings. We observed that cluster membership was largely defined by liquid-based food items (i.e. coffee, alcohol, sugary drinks), even after performing scaling procedures, leading us to separate food and beverage categories into two separate factor analyses. Using graphs generated via the *NbClust* v3.0 R package, we visually derived the number for ‘k’^(36)^. Energy contribution information from specific foods was not included in the clustering. For a further description of the food and nutrient intake across the derived clusters, we calculated the nutrient intake and mean intake of food from each food group.

Clusters derived from both dietary and meal pattern analyses in terms of their association with both categorical (e.g. sex) and continuous (e.g. BMI) variables using, respectively, Fisher’s exact test or the Tukey test. Differences between meal patterns derived via clustering in terms of nutrient and food group intake were identified via an ANOVA model as implemented by the *aov* function in R, using the Tukey post-hoc test to correct for multiple comparisons between clusters^(37)^.

Differences in energy, nutrient and food group intake, as well as participants’ clinical information relating to body composition and PAL, were also identified between dietary and meal patterns utilising the same method. Finally, for participants derived from each meal pattern cluster, we tested for any significant overlap with their dietary pattern cluster via enrichment analysis using Fisher’s exact test.

## Results

The results presented are from 192 participants with obesity who we recruited for a randomised controlled trial in Norway (the CARBFUNC study). Although BMI and fat mass being similar between sexes, the male participants had a significantly higher body weight and waist circumference compared with the female participants (online Supplementary Table S1).

### Dietary intake and physical activity level

As recording habitual dietary intake was a prerequisite for participation in the CARBFUNC study, the compliance was extraordinarily high. Dietary data collection could maximally give 1152 recording days, and the actual number of recording days was 1141, giving a completion percentage of 99·1% for all participants. 77 % of the participants reported 5 consecutive days with a 1-d gap to include a weekend day, while 19 % reported 6 consecutive days.

The recorded daily energy intake was not significantly different between females (8870 (sd 2176) kJ) and males (10 669 (sd 2301) kJ) (online Supplementary Table S2). The BMR was estimated to be non-significantly lower among females (7263 (sd 820) kJ) compared with male (8841 (sd 891) kJ) participants (online Supplementary Table S1). The ratio of energy intake to estimated BMR was 1·2 (sd 0·3) for both sexes, indicating a very low average PAL. The self-reported occupational and leisure-time physical activity showed, however, a higher average PAL of 1·5. PAL was significantly higher (95 % CI –0·17, –0·03, *P* = 0·004) in females (1·6 (sd 0·2)) than males (1·5 (sd 0·2)) (online Supplementary Table S1).

As expected, the male participants had a higher average intake of all macronutrients, contributing to the higher energy intake compared with the female participants (online Supplementary Table S2). The female participants had a non-significantly higher intake of added sugar, which is the only difference that becomes significant when adjusting for energy intake (95 % CI –20·7, –4·70, *P* = 0·02). Although mean alcohol consumption was 5 (sd 8) g/d, only half (48 %) of the participants reported alcohol consumption, resulting in an average intake of 10 (sd 9) g/d among the participants reporting alcohol consumption.

### Meal patterns derived from hierarchical cluster analysis

**Fig. 1.**
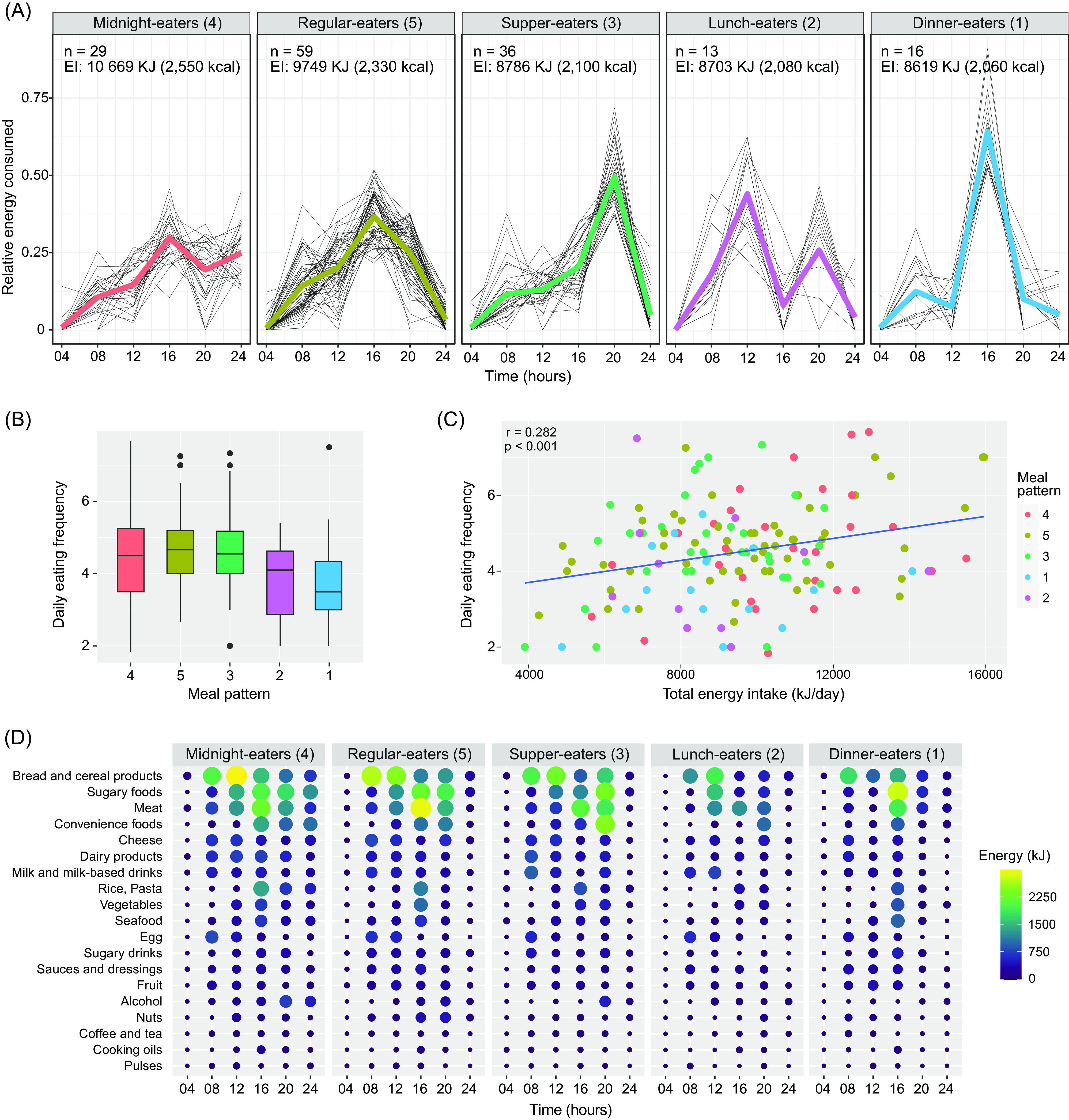
Meal patterns and associated eating frequencies and food groups. **A**: Relative energy intake for all six four-hour periods in the five meal patterns ordered from highest total energy intake (Midnight-eaters) to lowest (Dinner-eaters) from right to left. **B**: Box-whisker plots showing mean daily eating frequency of the five meal patterns, obtained by counting the number of time points of recorded dietary data for each participant throughout each of the six recording days. **C**: The relationship between mean daily eating frequency and reported total daily energy intake in the total study population, each point representing a participant. **D**: Energy intake from the fifteen food and beverage groups for every four-hour period in the five meal patterns. The brighter color and larger dots indicate higher energy contribution from a specific food or beverage group.

We extracted five non-overlapping meal patterns based on total energy intake during six 4-h periods (Fig. 1(a)), including both male (M) and female (F) participants, and named them according to the time-of-day with the highest peak in mean energy intake when comparing meal patterns: (1) dinner-eaters; (2) lunch-eaters; (3) supper-eaters; (4) midnight-eaters; and (5) regular-eaters. In total, 153 participants (F: 84/M: 67) were included in the meal pattern analysis, with 16 (F: 11/M: 5), 13 (F: 8/M: 5), 36 (F: 21/M: 15), 29 (F: 16/M: 13) and 59 (F: 29F/M: 30M) participants belonging to meal patterns 1–5, respectively. Participants without full and valid time records were excluded (*n* 39).

Overall, the midnight-eaters had the highest reported mean energy intake (10 669 (sd 2301) kJ), and significantly higher than dinner-eaters (8619 (sd 2301) kJ, 95 % CI 137·7, 835·0, *P* = 0·019), lunch-eaters (8703 (sd 2176) kJ, 95 % CI 102·2, 834·4, *P* = 0·007) and supper-eaters (8786 (sd 1925) kJ, 95 % CI 190·8, 705·9, *P* = 0·011), but not regular-eaters (9749 (sd 2720) kJ, 95 % CI −44·42, 489·0, *P* = 0·370) (Table 2). As the meal pattern clusters were based on all recordings for every 4 h combined, we also analysed frequency directly by counting each unique eating occasion throughout the day. The dinner-eaters showed the lowest eating frequency (3·56 (sd 1·04) times per day on average), which was significantly lower than regular-eaters (4·7 (sd 1·01), 95 % CI −2·64, −0·39, *P* = 0·013), lunch-eaters (4·0 (sd 1·53), 95 % CI −3·08, −0·39, *P* = 0·367) and supper-eaters (4·68 (sd 1·27), 95 % CI −2·55, −0·18, *P* = 0·026), but not the or the midnight-eaters (4·58 (sd 1·49), 95 % CI −1·37, 1·74, *P* = 0·068) (Fig. 1(b)). Eating frequency also correlated positively with total daily energy intake across the meal patterns (Fig. 1(c)).

**Table 2.** Mean energy intake (kJ) in total and during every 4-h period in the five meal patterns ordered from highest total energy intake (midnight-eaters) to lowest (dinner-eaters) (Mean values and standard deviation)

Meal pattern	04.00		08.00		12.00		16.00		20.00		00.00		Total energy	
	Mean	sd	Mean	sd	Mean	sd	Mean	sd	Mean	sd	Mean	sd	Mean	sd
Midnight-eaters (*n* 29)	75	310	1130	812	1565	929	3171	941	2075	1397	2657	1046	10 673	2318
Regular-eaters (*n* 59)	59	109	1389	925	1966	1151	3569	1084	2435	1197	326	485	9740	2732
Supper-eaters (*n* 36)	63	255	1017	933	1146	854	1799	879	4351	1381	427	732	8795	1929
Lunch-eaters (*n* 13)	0	0	1577	1536	3833	1891	699	941	2247	1837	356	623	8711	2602
Dinner-eaters (*n* 16)	63	615	1079	983	653	925	5552	1527	858	845	435	623	8636	2284

Comparing each 4-h period throughout the day, the energy intake during the first two periods of the day did not significantly differ between the meal patterns. During the early morning (04.00 ± 2 h), the energy intake reported was clinically insignificant for all meal patterns, while energy intake at breakfast (08.00 ± 2 h) ranged from 1017 to 1577 kJ (Table 2). For lunchtime (12.00 ± 2 h), the mean energy intake varied from 3833 kJ for the lunch-eaters compared with 669–1966 kJ in the other meal patterns. The largest variation between the meal patterns was at dinnertime between the lunch-eaters and dinner-eaters, consuming on average 711 and 5565 kJ, respectively. The supper-eaters consumed 4351 kJ on average in the evening compared with 879–2427 kJ in the other meal patterns. During the last period of the day, the energy intake of the midnight-eaters (2657 (sd 1046) kJ) was significantly higher compared with the four remaining meal patterns (regular-eaters: 95 % CI 288·3, 684·1, *P* < 0·001; supper-eaters: 95 % CI 318·7, 718·6, *P* < 0·001; lunch-eaters: 95 % CI 454·4, 849·8, *P* < 0·001; dinner-eaters: 95 % CI 334·0, 758·3, *P* < 0·001).

Further, we compared energy intake from each of the fifteen food and beverage groups for every 4-h period between the meal patterns (Fig. 1(d)). The midnight-eaters with the overall highest energy intake (10 669 (sd 2301) kJ) had the highest mean intake of alcohol, oil, rice, pasta and sugary foods compared with the other meal patterns (online Supplementary Fig. S1). The highest mean intake of fish, fruit and vegetables was found in the dinner-eaters with the overall lowest energy intake (8619 (sd 2301) kJ). The lunch-eaters consumed the highest amounts of bread and cereal products, cheese, pulses, milk and milk-based drinks (online Supplementary Fig. S1). However, these differences in specific food group intakes were not statistically significant between the meal patterns.

Despite a difference of 2050 kJ in reported energy intake (rEI) between the midnight-eaters and the dinner-eaters (lowest energy intake), we did not find any significant differences in the anthropometric measures including body weight, BMI and waist circumference.

### Dietary patterns derived from k-means cluster analysis

**Fig. 2.**
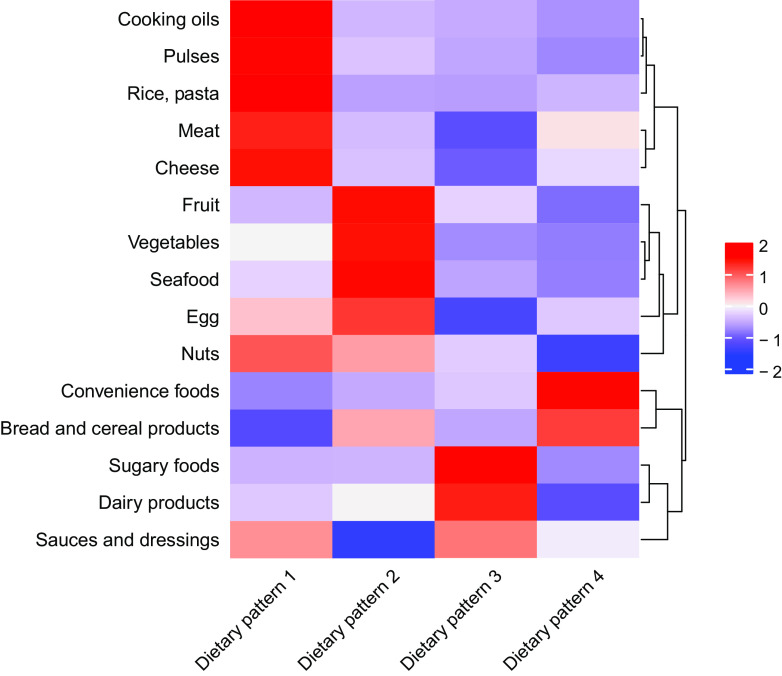
Dietary patterns and their defining food groups. Heat map and k-means clustering showing the four dietary patterns and the fifteen food groups. The colors correspond to the mean intake of each food group in the four dietary patterns compared to the overall mean intake in the study population (red: above mean intake; white: at mean intake; blue: below mean intake). The four dietary patterns are named according to their defining food groups: 1: Meat, rice, pasta, pulses and oil, 2: Vegetables, fruit, and seafood, 3: Sugary foods, 4: Bread, cereal products, and convenience foods.

Including both female and male participants, we extracted four non-overlapping dietary patterns based on the variation of the mean daily intake in g of the food and beverage groups (Fig. 2). These patterns were named according to the food groups characterising each of them: (1) meat, rice, pasta, pulses and oil; (2) vegetables, fruit and seafood; (3) sugary foods; and (4) bread, cereal products and convenience foods. The number of participants was 39 (F: 19/M: 20), 44 (F: 26/M: 18), 55 (F: 36/M: 19) and 54 (F: 20/M: 34) for dietary patterns 1–4, respectively.

**Fig. 3.**
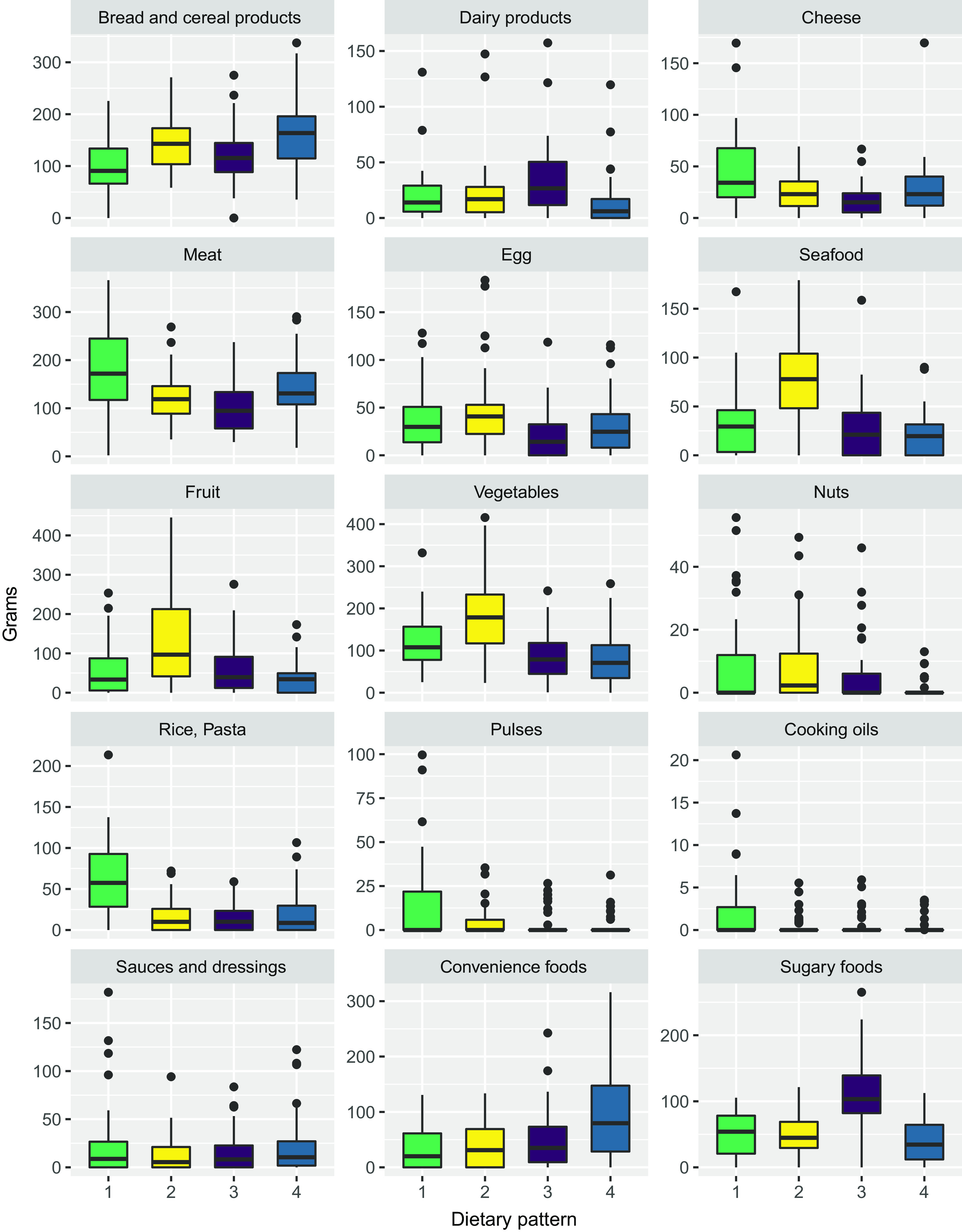
Box-whisker plots showing the mean intake (in grams) of the fifteen food groups in the four dietary patterns. The different colors correspond to the four different dietary patterns: pattern 1: green; pattern 2: yellow; pattern 3: purple; pattern 4: blue. The dietary patterns are named according to their defining food groups: pattern 1: Meat, rice, pasta, pulses, and oil; pattern 2: Vegetables, fruit, and seafood; pattern 3: Sugary foods; pattern 4: Bread, cereal products, and convenience foods.

In dietary pattern 1 (meat, rice and pasta, pulses and oil), the intake of each of the four food groups was significantly higher compared with dietary patterns 2–4 (online Supplementary Table S3). Meat intake in dietary pattern 1 was on average 180 g/d (1250 g/week) and was 30 % higher compared with dietary pattern 4 with the second highest meat intake of 140 g/d (980 g/week) (Fig. 3).

In dietary pattern 2 (vegetables, fruit and seafood), the intake of each of the three food groups was significantly higher compared with dietary patterns 1 and 3–4 (online Supplementary Table S4). Although non-significant, fibre intake was highest in dietary patterns 2 and 4 (24 g/d) compared with dietary patterns 1 and 3 (20 g/d).

In dietary pattern 3 (sugary foods), the intake of sugary foods was significantly higher compared with dietary patterns 1–2 and 4 (online Supplementary Table S5). The intake of 110 g/d of sugary foods was over twice as high as in dietary pattern 2 with the second highest intake of 50 g/d. The mean intake of added sugar was 56 g. This was significantly higher compared with each of the remaining dietary patterns, and 50 % higher compared with dietary pattern 4 with the second highest intake of added sugar (Fig. 3). Mean carbohydrate intake was significantly higher for dietary patterns 3 (253 g) and 4 (247 g) compared with dietary patterns 1 (200 g) and 2 (209 g).

In dietary pattern 4 (bread, cereal products and convenience foods), the intake of convenience foods was significantly higher compared with dietary patterns 1–3, while the intake of bread and cereal products was only significantly higher compared with dietary patterns 1 and 3, not 2 (online Supplementary Table S6).

Mean energy intake was highest in dietary pattern 3 (10 167 (sd 2473) kJ), followed by dietary pattern 4 (10 104 (sd 2569) kJ), dietary pattern 1 (9288 (sd 2276) kJ) and dietary pattern 2 (9079 (sd 2033) kcal). Although there were significant differences in the intake of food groups, the differences in energy intake were not significant.

## Discussion

In the present exploratory analysis, we analysed specific dietary and meal patterns in community-dwelling adults with obesity, to gain deeper insight into the diversity of typical dietary habits. By including several approaches to examine the habitual diet, we identified five meal patterns by time-of-intake of daily energy consumption, and four dietary patterns derived from the intake of specific food groups. Specific meal patterns, and to a lesser extent dietary patterns, corresponded to differences in total daily energy intake.

### Meal timing and total energy intake

A primary finding in our meal pattern analysis is that participants with the highest energetic intake around midnight had the highest overall daily energy intake (10 669 kcal/d), corresponding to more frequent eating. These findings are partly consistent with previous studies suggesting that, in adults with BMI within the normal range, consuming a high proportion of daily energy intake at night or late evening is associated with higher total energy intake^(38,39)^. On the other hand, these studies found that a higher intake in the morning or afternoon was associated with a lower total energy intake, which is only partially in agreement with the current study. In the present study, although the dinner-eaters with the lowest overall energy intake reported consuming the highest proportion of their energy intake in the afternoon, we found no association between energy intake in the morning, for example, eating breakfast (08.00 ± 2 h), and total energy intake.

### Intake of food and beverage groups among meal patterns

Our data reveal intake of specific food and beverage groups for different meal patterns. Intake of foods with relatively low energy density and glycaemic index, such as fruits and vegetables, was highest in the dinner-eaters with the overall lowest reported daily energy intake. Conversely, the consumption of energy-dense foods and beverages such as alcoholic beverages and foods with higher glycaemic index, for example, sugary foods and pasta, was highest among the midnight-eaters, likely contributing to higher glycaemic load as well as overall energy intake in this group. Differences in energy density might at least partly explain the different total energy intakes^(40)^. Of note, although alcohol consumption contributed to the overall higher energy intake in the midnight-eaters, the differences in alcohol consumption between the meal patterns were small. This may partially be explained by the exclusion of participants with an alcohol consumption of > 2 alcohol units per day from our study.

### Dietary patterns and associated food groups and energy intake

Unlike the results for meal patterns, we did not find significant differences in energy intake between dietary patterns. Nonetheless, the difference in mean energy intake between the two dietary patterns ‘sugary foods’ and ‘vegetables, fruit and seafood’ was 11 %, which may be clinically relevant. When comparing intake of specific food groups between the dietary pattern and the meal pattern with the overall lowest energy intakes (the vegetables, fruit and seafood pattern and the dinner-eaters), we found that both patterns had the highest mean intake of vegetables, fruit and seafood compared with the other dietary and meal patterns. As for the midnight-eaters in the meal pattern analysis, the daily intake of sugary foods was highest in the dietary pattern with the overall highest energy intake (sugary foods pattern). In agreement with our findings, dietary patterns dominated by sugary foods have previously been associated with the highest overall energy intake^(41)^.

### Differences in reported energy intake and anthropometric measures

Although differences in reported mean energy intake accounted for up to 2050 kJ/d between the clusters in the meal pattern analysis, we found no difference in anthropometric measures including waist circumference and body weight. We also found no differences in estimated BMR using the Mifflin St Jeor predictive equation nor in reported PAL (self-reported and leisure-time physical activity) between clusters. In addition, possible confounders including age and distribution of male and female participants did not differ between clusters. In a Swedish study comparing eating patterns in people with normal weight or overweight (BMI < 30) and with obesity (BMI ≥ 30), no significant differences were found in overall energy intake, even after adjusting for PAL^(42)^. However, the authors found an association between obesity and both night-time meals and portion sizes, even though there were no differences in rEI. A recent study investigating meal timing and BMI dependent on chronotype found on average only a 728 kJ difference in mean daily energy intake when comparing normal-weight subjects (8322 kcal) and subjects with obesity (9050 kcal)^(43)^.

Underestimation of actual intake and under-reporting among adults with obesity is a well-described phenomenon^(44,45)^, and this might partly explain the non-significant differences in self-rEI between normal-weight subjects and subjects with obesity in previous studies. To further explore whether the differences in self-rEI could be explained by differences in the level of under-reporting in our study, we used both recommended cut-off levels (lower cut-off < 2092 and < 3347 kJ (< 500 and < 800 kcal), upper cut-off >14 644 and >16 736 kJ (> 3500 and > 4000 kcal) for women and men, respectively) and the Goldberg cut-off, revealing no significant differences in the estimated level of under- or over-reporting between clusters. These data suggest that the differences in rEI between meal patterns cannot be attributed to under-reporting being overrepresented in any of the clusters. We cannot rule out that the lack of association between total energy intake and anthropometric measures was due to differences in biological regulatory mechanisms that were not measured in the present study, including circadian, thermogenic, hormonal and other effects of the different meal and dietary patterns.

Overall, our data highlight the potential importance of identifying a person’s habitual meal pattern, beyond general dietary pattern, to inform more precise interventions aligned with a person’s specific sources of excess energy intake. Additionally, awareness that groups of people with obesity can have meal patterns with relatively lower energy intakes, as observed in our study, may help to gain a more nuanced understanding of the different biological as well as lifestyle factors that promote or maintain obesity in a given individual.

### Strengths and limitations

A strength of our study is the detailed real-time dietary data obtained using multiple consecutive days of weighed dietary records to account for day-to-day variability in energy intake and eating behaviours^(46)^. Furthermore, our database was manually curated to include all food and beverage items consumed during the study period. This resulted in a comprehensive database adapted for the current study. Also, all recordings were manually quality controlled by trained personnel and the recording completion rate was high (99 %). To our knowledge, no previous studies have explored the habitual diet, with the inclusion of data on nutrient intake, dietary patterns and meal patterns, in a healthy, weight-stable, non-smoking, population with obesity.

Generalisability of our findings is, however, limited as our study participants were recruited for a weight-loss trial and are not necessarily representative of the general population with obesity. Overall, comparing our findings of meal patterns with previous relevant studies is challenging for several reasons. The use of different defining criteria for meals is widespread, including meals defined by time-of-day^(47)^, timing relative to individual sleep/wake timing^(43)^ and self-identified eating occasions as a snack or a meal^(48)^. Also, differences in categorisation of time-of-day occur, including dividing 24 h into morning, midday and evening^(49)^, morning, afternoon and night^(38)^, or into periods of varying duration and number, as in 24 h divided into five 4-h periods excluding 02·00–05·59^(39)^, or six 4-h periods as in the current study. We cannot conclude that our approach is superior to other defining criteria.

Among the limitations of this study is the lack of an a priori power calculation to assess adequate sample size to detect possible differences in the reported outcome measures. Power calculation based on the primary outcome from the dietary trial was conducted a priori but not for the secondary outcomes included in this cross-sectional analysis. We chose not to conduct post-hoc analyses as this approach is considered flawed^(50)^. However, we did observe a statistically significant difference in energy intake between meal pattern clusters, supporting sufficient power.

### Conclusions

In conclusion, our data reveal five distinct meal patterns in a population with obesity, and a clear relationship between daily meal patterns, eating frequency and total energy intake, although no correlations with anthropometric measures were found. Our data support that the identification of individual meal patterns may facilitate the clinical application of more personalised prevention and treatment strategies. For example, identification of a ‘midnight-eater’ or a ‘supper-eater’ can allow targeted, time-specific adjustments of the person’s food-, beverage- and energy intake, beyond general advice to reduce energy intake throughout the whole day. Our observations encourage further research, in particular prospective studies, to explore whether changes in meal patterns and daily distribution of energy intake can lead to both beneficial changes in energetic intake and anthropometric measures in people with obesity.

## Supplementary material

For supplementary material accompanying this paper visit https://doi.org/10.1017/S0007114521002580. click here to view supplementary material
