# Supplementary material for: Meal patterns associated with energy intake in people with obesity
Source: Br J Nutr. 2021 Jul 12;128(2):334–44. doi: 10.1017/S0007114521002580 (PMC9301523; doi:10.1017/S0007114521002580)
Supplement: Supplementary file 1 [file S0007114521002580sup.zip › S0007114521002580sup001.docx]

**Supplementary Table 2**

Reported energy intake and contribution from macronutrients according to sex and meal pattern¹

|  | All *(n = 192)* | Female  (*n* = 101) | Male  (*n* = 91) | Midnight-eaters 4 *(n = 29)* | Regular-eaters 5 *(n = 59)* | Supper-eaters 3 *(n = 36)* | | | Lunch-eaters 2 *(n = 13)* | | Dinner-eaters 1 *(n = 13)* | |  |  |  |  |
| --- | --- | --- | --- | --- | --- | --- | --- | --- | --- | --- | --- | --- | --- | --- | --- | --- |
| Energy |  |  |  |  |  |  | | |  | |  | |  |  |  |  |
| kJ | 9710 ± 2380 | 8860 ± 2160 | 10670 ± 2310^2^ | 10600 ± 2290 | 9600 ± 2790 | 8710 ± 1890 | | | 8620 ± 2120 | | 8570 ± 2200 | |  |  |  |  |
| kcal | 2320 ± 570 | 2120 ± 520 | 2550 ± 550 | 2551 ± 554 | 2328 ± 653 | 2102 ± 461 | | | 2082 ± 522 | | 2064 ± 546 | |  |  |  |  |
| Protein |  |  |  |  |  |  | | |  | |  | |  |  |  |  |
| grams | 101 ± 28 | 89 ± 25 | 114 ± 25^2^ | 106 ± 28 | 98 ± 25 | 100 ± 33 | | | 98 ± 35 | | 91 ± 24 | |  |  |  |  |
| kJ | 1690 ± 469 | 1490 ± 418 | 1908 ± 418 | 1774 ± 464 | 1640 ± 414 | 1665 ± 544 | | | 1632 ± 586 | | 1531 ± 406 | |  |  |  |  |
| Carbohydrate |  |  |  |  |  |  | | |  | |  | |  |  |  |  |
| grams | 231 ± 70 | 215 ± 64 | 247 ± 72^2^ | 247 ± 73 | 230 ± 81 | 211 ± 53 | | | 207 ± 48 | | 204 ± 78 | |  |  |  |  |
| kJ | 3866 ± 1172 | 3598 ± 1071 | 4134 ± 1205 | 4134 ± 1218 | 3849 ± 1356 | 3531 ± 883 | | | 3464 ± 803 | | 3414 ± 1310 | |  |  |  |  |
| Fiber |  |  |  |  |  |  | | |  | |  | |  |  |  |  |
| grams | 22 ± 11 | 20 ± 7 | 24 ± 13^2^ | 22 ± 6 | 22 ± 9 | 21 ± 9 | | | 22 ± 7 | | 19 ± 8 | |  |  |  |  |
| kJ | 184 ± 92 | 167 ± 59 | 205 ± 109 | 180 ± 50 | 180 ± 75 | 176 ± 75 | | | 184 ± 59 | | 159 ± 67 | |  |  |  |  |
| Added sugar |  |  |  |  |  |  | | |  | |  | |  |  |  |  |
| grams | 38 ± 29 | 39 ± 28 | 37 ± 30 | 37 ± 21 | 41 ± 36 | 32 ± 26 | | | 29 ± 21 | | 44 ± 41 | |  |  |  |  |
| kJ | 636 ± 485 | 644 ± 469 | 623 ± 502 | 628 ± 351 | 682 ± 602 | 536 ± 435 | | | 494 ± 343 | | 736 ± 678 | |  |  |  |  |
| Fat |  |  |  |  |  |  | | |  | |  | |  |  |  |  |
| grams | 104 ± 35 | 93 ± 32 | 116 ± 34^2^ | 117 ± 40 | 106 ± 37 | 90 ± 26 | | | 90 ± 39 | | 92 ± 32 | |  |  |  |  |
| kJ | 3916 ± 1318 | 3506 ± 1205 | 4351 ± 1280 | 4406 ± 1506 | 3992 ± 1385 | 3389 ± 979 | | | 3381 ± 1460 | | 3481 ± 1197 | |  |  |  |  |
| SFA |  |  |  |  |  |  | | |  | |  | |  |  |  |  |
| grams | 38 ± 16 | 34 ± 14 | 43 ± 16^2^ | 45 ± 19 | 38 ± 15 | 33 ± 12 | | | 31 ± 16 | | 33 ± 15 | |  |  |  |  |
| kJ | 1431 ± 602 | 1280 ± 527 | 1607 ± 602 | 1678 ± 715 | 1448 ± 565 | 1259 ± 448 | | | 1184 ± 586 | | 1247 ± 561 | |  |  |  |  |
| MUFA |  |  |  |  |  |  | | |  | |  | |  |  |  |  |
| grams | 33 ± 12 | 30 ± 11 | 38 ± 12^2^ | 36 ± 13 | 35 ± 13 | 29 ± 10 | | | 31 ± 16 | | 32 ± 12 | |  |  |  |  |
| kJ | 1243 ± 452 | 1113 ± 414 | 1414 ± 452 | 1343 ± 494 | 1301 ± 498 | 1092 ± 360 | | | 1159 ± 586 | | 1209 ± 452 | |  |  |  |  |
| PUFA |  |  |  |  |  |  | | |  | |  | |  |  |  |  |
| grams | 13 ± 6 | 12 ± 6 | 15 ± 5^2^ | 14 ± 6 | 14 ± 6 | 12 ± 4 | | | 13 ± 7 | | 14 ± 5 | |  |  |  |  |
| kJ | 490 ± 226 | 464 ± 226 | 548 ± 188 | 544 ± 218 | 510 ± 238 | 435 ± 146 | | | 494 ± 259 | | 527 ± 197 | |  |  |  |  |
| Cholesterol |  |  |  |  |  |  | | |  | |  | |  |  |  |  |
| grams | 0.3 ± 0.2 | 0.3 ± 0.2 | 0.4 ± 0.2^2^ | 0.4 ± 0.2 | 0.3 ± 0.2 | 0.3 ± 0.2 | | | 0.3 ± 0.3 | | 0.3 ± 0.1 | |  |  |  |  |
| kJ | 13 ± 8 | 13 ± 8 | 13 ± 8 | 13 ± 4 | 8 ± 4 | 8 ± 4 | | | 8 ± 8 | | 8 ± 4 | |  |  |  |  |
| Alcohol |  |  |  |  |  |  | | |  | |  | |  |  |  |  |
| grams | 5 ± 8 | 4 ± 7 | 6 ± 9 | 8 ± 11 | 4 ± 7 | 4 ± 7 | | | 5 ± 5 | | 2 ± 3 | |  |  |  |  |
| kJ | 146 ± 234 | 117 ± 205 | 167 ± 264 | 226 ± 314 | 117 ± 205 | 117 ± 201 | | | 134 ± 155 | | 54 ± 75 | |  |  |  |  |
| ¹ Values are means ± SDs.  ^2^ Significantly different (*P* < 0.05) from female participants (non-paired t-test). MUFA, monounsaturated fatty acids; PUFA, polyunsaturated fatty acids; SFA, saturated fatty acids. | | | | | | |  |  | |  | |  | |  |  |  |
