# Supplementary figures and images for: Meal patterns associated with energy intake in people with obesity
Source: Br J Nutr. 2021 Jul 12;128(2):334–44. doi: 10.1017/S0007114521002580 (PMC9301523; doi:10.1017/S0007114521002580)

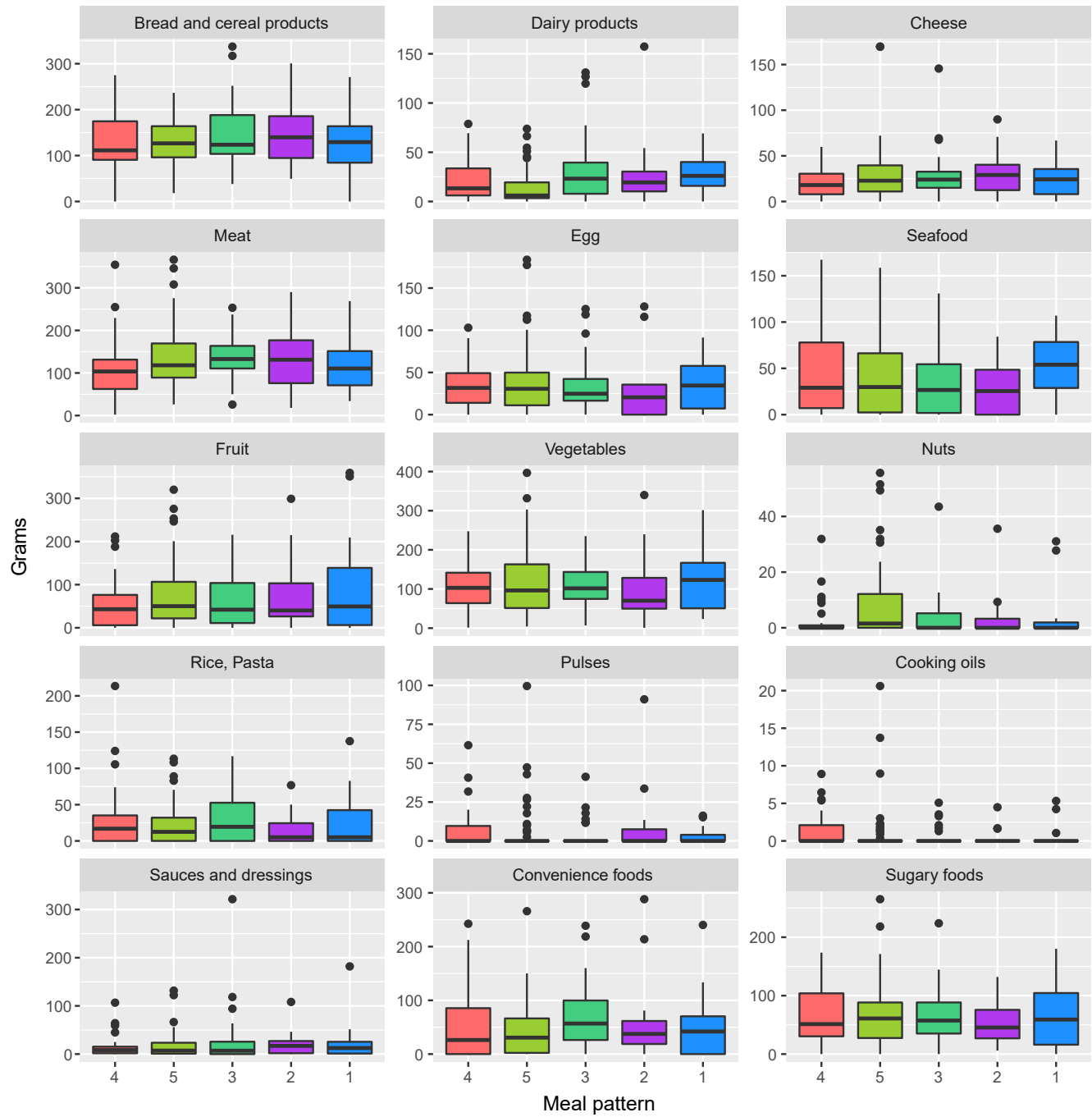

Supplement: Supplementary file 1 [file S0007114521002580sup.zip › S0007114521002580sup002.pdf]
